# A quantitative shRNA screen identifies *ATP1A1* as a gene that regulates cytotoxicity by aurilide B

**DOI:** 10.1038/s41598-017-02016-4

**Published:** 2017-05-17

**Authors:** Shohei Takase, Rumi Kurokawa, Daisuke Arai, Kind Kanemoto Kanto, Tatsufumi Okino, Yoichi Nakao, Tetsuo Kushiro, Minoru Yoshida, Ken Matsumoto

**Affiliations:** 1 Chemical Genomics Research Group, RIKEN Center for Sustainable Resource Science, Saitama, 351-0198 Japan; 2 School of Agriculture, Meiji University, 1-1-1 Higashimita, Tama-ku, Kawasaki, Kanagawa 214-8571 Japan; 3 Chemical Genetics Laboratory, RIKEN, Saitama, 351-0198 Japan; 4 Research Institute for Science and Engineering, Waseda University, 3-4-1 Okubo, Shinjuku-ku, Tokyo 169-8555 Japan; 5 College of Micronesia - FSM Chuuk Campus, P.O. Box 879, Chuuk, 96942 Federated States of Micronesia; 6 Faculty of Environmental Earth Science, Hokkaido University, Sapporo, 060-0810 Japan; 7 Seed Compounds Exploratory Unit for Drug Discovery Platform, Drug Discovery Platforms Cooperation Division, RIKEN Center for Sustainable Resource Science, Saitama, 351-0198 Japan; 8 Japan Agency for Medical Research and Development, AMED-CREST, Tokyo, Japan

## Abstract

Genome-wide RNA interference (RNAi) with pooled and barcoded short-hairpin RNA (shRNA) libraries provides a powerful tool for identifying cellular components that are relevant to the modes/mechanisms of action (MoA) of bioactive compounds. shRNAs that affect cellular sensitivity to a given compound can be identified by deep sequencing of shRNA-specific barcodes. We used multiplex barcode sequencing technology by adding sample-specific index tags to PCR primers during sequence library preparation, enabling parallel analysis of multiple samples. An shRNA library screen with this system revealed that downregulation of ATP1A1, an α-subunit of Na^+^/K^+^ ATPase, conferred significant sensitivity to aurilide B, a natural marine product that induces mitochondria-mediated apoptosis. Combined treatment with ouabain which inhibits Na^+^/K^+^ ATPase by targeting α-subunits potentiated sensitivity to aurilide B, suggesting that ATP1A1 regulates mitochondria-mediated apoptosis. Our results indicate that multiplex sequencing facilitates the use of pooled shRNA library screening for the identification of combination drug therapy targets.

## Introduction

Target identification of bioactive compounds is a long-standing challenge for drug development, as well as for chemical biology studies aimed at elucidating cellular functions using compounds. Cell-based assays based on disease-specific phenotypes, such as cell proliferation, migration, and invasion in the case of cancers, have been used to identify lead compounds^1, 2^. However, elucidation of the MoA and molecular targets of compounds obtained by phenotypic screening remains challenging, and is therefore a rate-limiting step for drug discovery using cell-based assays. The combination of affinity-based approaches, knowledge-based profiling approaches, and genetic approaches has proven to be a powerful means of gaining insight into compound MoA^2^. In the context of chemical genetics, genome-wide RNAi technology in mammalian cultured cells is considered to be an unbiased comprehensive strategy. Screening of genome-wide pooled shRNA libraries enables identification of cellular components that are functionally relevant to the MoA of a compound, as well as its direct target(s)^2–6^. Differences between control and compound-treated cell populations in the growth rates of cells carrying a specific shRNA can be detected by deep sequencing, and this approach has a broader dynamic range than DNA microarrays. Despite this advantage, this type of screening is costly and low-throughput, and it would therefore be helpful to improve its throughput and cost-efficiency.

The cyclodepsipeptide aurilide B, originally isolated from the marine cyanobacterium *Lyngbya majuscule*, is closely related to the cytotoxic compound aurilide from the sea hare *Dolabella auricularia* ^7–10^. Aurilide binds to prohibitin 1 in mitochondria, thereby activating the processing of optic atrophy 1 (OPA1) and leading in turn to mitochondrial fragmentation and apoptosis^11^. Aurilide B exerts potent cytotoxicity in human leukemia, renal, and prostate cancer cell lines, with a GI_50_ less than 10 nM^10^. Therefore, aurilide and aurilide B are considered to have therapeutic potential, prompting us to identify potential combinatorial drug targets by screening for a gene(s) whose downregulation sensitizes cells to these compounds.

In this study, we introduced a strategy for increasing the throughput of shRNA identification after screening with pooled and barcoded shRNA libraries. The addition of a sample-specific index tag to a PCR primer during sequence library preparation enabled sorting of samples analyzed in parallel by deep sequencing. We show that multiplex barcode sequencing is a reproducible, quantitative, and feasible approach for identifying molecular targets of bioactive compounds. We then used our method to identify genes that modify cellular sensitivity to aurilide B. The results revealed *ATP1A1*, which encodes a catalytic α-subunit of Na^+^/K^+^ ATPase, as a gene involved in survival of cells treated with aurilide B. Indeed, ouabain, a known inhibitor that binds to α-subunits of Na^+^/K^+^ ATPase^12, 13^, potentiated the cytotoxicity of aurilide B. This study provides not only a proof of concept for the use of genome-wide shRNA library screening to identify unexpected indirect target genes of bioactive compounds, but also a novel mechanism underlying aurilide B–induced cancer cell death.

## Results and Discussion

### Multiplex barcode sequencing following shRNA library screens

We used the Decipher lentiviral and pooled shRNA libraries to identify genes involved in MoA or synthetic-lethal pathways of a compound of interest. Decipher libraries targeting ~15,000 human genes consist of three modules (Human Modules 1, 2, and 3), each of which contains five or six shRNAs for ~5,000 independent genes. Each shRNA construct harbors a specific barcode of 18 nucleotides (nt) adjacent to the shRNA sequence that facilitates subsequent identification of shRNAs. Following the shRNA screen, barcode regions are amplified by PCR using primers specific for the vector sequences, and the products are subjected to next-generation sequencing. However, due to constraints in the vector structures, barcode analyses of cell populations transduced with each module needed to be performed independently in each lane of a next-generation sequencer, creating an obstacle to high-throughput experiments. In order to increase the throughput of barcode sequencing after shRNA screening, we established a strategy for multiplex barcode sequencing by adding 8-nt index sequences to the PCR primers used for barcode amplification, allowing us to distinguish each sample in the same analysis (see Supplementary Fig. S1). Currently, we analyze four samples together in a single Illumina HiSeq2500 lane in order to maintain a minimum read count of 20 million per sample.

**Figure 1:**
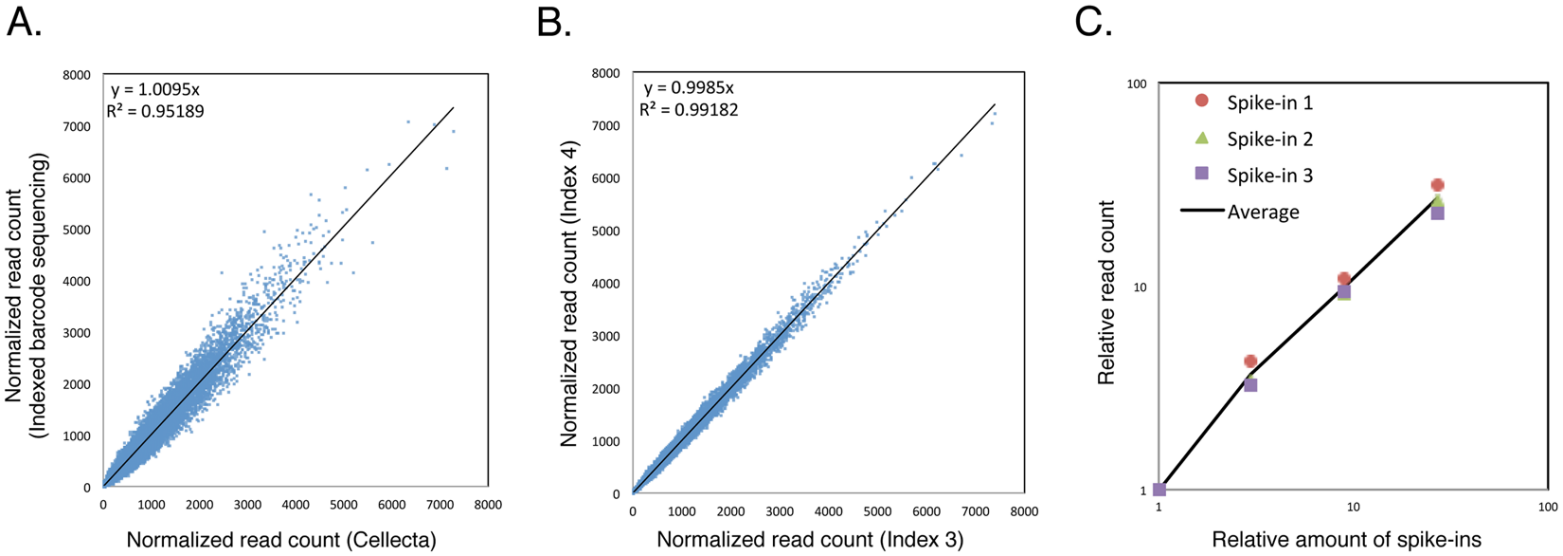
Reproducible and quantitative barcode amplification with indexed primers. (**A**) HeLa S3 cells transduced with Human Module 1 library were selected with puromycin for 2 days, cultured without the test compounds for an additional 10 days, and then harvested. An aliquot of the cells was sent to Cellecta for DNA extraction, PCR amplification without index tags, and barcode quantitation by deep sequencing. For index-tagged barcode amplification, genomic DNA was isolated from another aliquot of cells, and the barcode regions were amplified. Different index tags were added by the second PCR step, as described in Experimental Procedures and Supplementary Fig. S1. Four samples were combined in a single deep sequencing lane, and barcode sequencing was performed. Normalized read counts of each barcode from one of the samples (y-axis) were compared with those obtained from independent sequencing by Cellecta (x-axis). (**B**) HeLa S3 cells transduced with the Human Module 3 library were cultured, and barcode sequencing with index tags was performed as in (**A**). Normalized read counts of each barcode from sequencing performed with two different index tags were compared with each other. (**C**) HeLa S3 cells transduced with the Human Module 1 library were cultured as in (**A**), and genomic DNA was isolated. Increasing amounts of three DNA spike-ins harboring designed barcodes were added to genomic DNA prior to PCR amplification. Normalized read counts of the designed barcodes were plotted against the amounts of spike-ins added. Averages were calculated using three spike-ins.

To validate that our modified technique enabled reproducible and quantitative amplification and faithfully reproduced barcode sequencing results obtained with unindexed primers, we first sought to determine whether the read counts of barcodes were affected by the addition of index sequences. To do this, we infected HeLa S3 cells with Human Module 1 and cultured the infected cells for 10 days. Then we subjected one aliquot to barcode amplification with indexed primers and analyzed another aliquot by conventional barcode amplification (Fig. 1A). Comparison of barcode read counts revealed no significant difference between the two experimental procedures (see Supplementary Table S1). Second, we compared the read counts of barcodes amplified with two different indexed primers and analyzed in different lanes in a next-generation sequencer. Again, the data revealed no appreciable changes in read counts between different index sequences (Fig. 1B). These results indicate that our modified PCR method is reproducible and suitable for multiplex barcode sequencing. Third, we assessed whether our PCR conditions amplified barcode regions quantitatively, using DNA spike-ins that were added to genomic DNA prior to amplification by PCR. We used three spike-ins harboring designed barcode sequences different from those found in the shRNA library (see Supplementary Table S2). The barcode read counts of DNA spike-ins were increased in a dose-dependent manner, indicating that our method works quantitatively (Fig. 1C). Taken together, these results illustrate the suitability of our multiplex barcode sequencing for analysis of shRNA library screens.

### shRNA screen with etoposide

**Figure 2:**
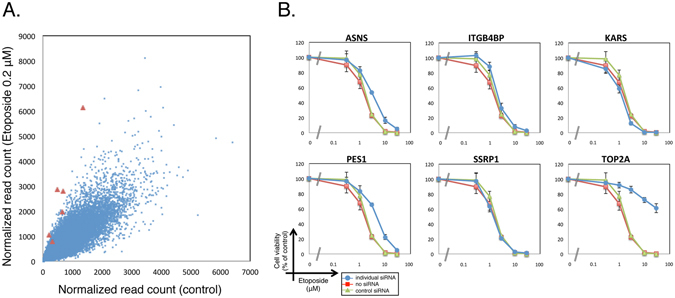
Validation of shRNA library screening using etoposide. (**A**) HeLa S3 cells were infected with Module 1 and split into two subpopulations, one of which was treated with 0.2 μM etoposide for 6 days. Relative read counts of each barcode were compared between treated and untreated cells. shRNAs against topo IIα are highlighted in red. (**B**) HeLa S3 cells were transfected with siRNA pools against 6 candidate genes, and then cultured for 2 days in the presence of the indicated concentrations of etoposide. Cell viability was measured by WST-8 assay. Data represent means ± SD from three independent experiments.

Next, to determine whether our shRNA library screen could identify genes that confer resistance or sensitivity to treatment with a compound of interest, we conducted shRNA library screening with etoposide which inhibits DNA topoisomerase II (topo II)^14, 15^. HeLa S3 cells were infected with Human Module 1, and the infected cells were split into two subpopulations, one of which was treated with 0.2 μM etoposide for 6 days. shRNA-specific barcodes were amplified from etoposide-treated and untreated cells using index-tagged PCR primers as described above, and the PCR products were subjected to deep sequencing. The normalized read counts of each barcode were compared between treated and untreated cells. As shown in a scattered plot, shRNAs against topo IIα were significantly enriched in treated cells (Fig. 2A), consistent with previous results obtained using The RNAi Consortium (TRC) shRNA library^16^.

The normalized read counts of each barcode from etoposide-treated cells were divided by those from untreated cells to obtain a fold change (etoposide-treated/untreated). All of six shRNAs against topo IIα were enriched with the fold change >2. To validate the shRNA screen results, we tested if topo IIα knockdown by a siRNA pool confers resistance to etoposide. As we obtained 5 other genes against which 5 or 6 shRNAs were enriched with the fold change >2 in the shRNA screen, we included these genes into the test with siRNA pools (Fig. 2B). To do this, cells were transfected with siRNA pools against the individual genes. After 48 h, transfected cells were split and seeded in 96-well plates, and then treated with different concentrations of etoposide for 48 h. Among the 6 genes, the knockdown of topo IIα significantly affected the sensitivity of cells to etoposide (Fig. 2B). Etoposide binds to topo II after the formation of a DNA double-strand break and inhibits the re-ligation of the topo II cleavage complex^15^. Therefore, in topo II–depleted cells, etoposide cannot induce DNA breaks, and the cells can survive. Overall, these results indicate that shRNA screens with bioactive compounds that affect the cell growth allow us to identify genes involved in their MoA.

### shRNA screen with aurilide B

**Figure 3:**
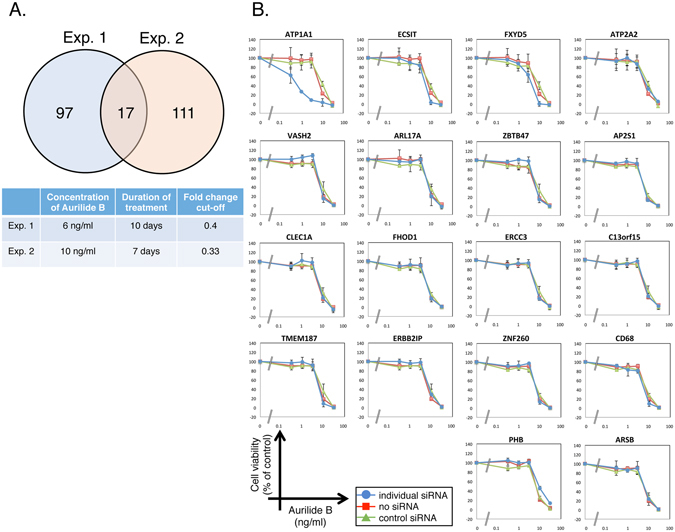
Candidate genes that determine the sensitivity to aurilide B. (**A**) shRNA screening for genes that modify the sensitivity to aurilide B was performed in HeLa S3 cells under two independent conditions. Seventeen genes whose knockdown sensitized cells to aurilide B were identified in both experiments (*P* = 4.07 × 10^−15^, Fisher’s exact test, R version 3.3.2). (**B**) HeLa S3 cells were transfected with siRNA pools against 17 candidate genes, and then cultured for 2 days in the presence of the indicated concentrations of aurilide B. Cell viability was measured by WST-8 assay. Data represent means ± SD from three independent experiments.

Next, we tried to search for genes that modify sensitivity to another target-predicted compound, aurilide B. Its close analogue, aurilide, binds to prohibitin 1 to induce mitochondria-mediated apoptosis^11^. In our shRNA screening, we targeted ~15,000 human genes using the Human Module 1, 2 and 3 in separate infections, and treated the infected cells with aurilide B under two independent conditions. To do this, we first infected HeLa S3 cells with the Human Module 1 and treated them with 6 ng/ml aurilide B for 10 days (Experiment 1) or 10 ng/ml aurilide B for 7 days (Experiment 2) (see Supplementary Fig. S2). The same experiments were repeated with Module 2-infected cells and Module 3-infected cells. The barcodes were amplified from aurilide B-treated and untreated cells using index-tagged PCR primers as described above, and the PCR products were subjected to deep sequencing. We wanted not only to confirm the MoA of aurilide B by shRNA screening, but also to search for genes related to synthetic lethality with aurilide B. Cells harboring shRNAs against candidate synthetic-lethal genes are expected to be depleted in aurilide B–treated cells in comparison with untreated cells. Accordingly, we selected genes with two or more shRNAs with a fold change (aurilide B-treated/untreated) <0.4 (Experiment 1) or 0.33 (Experiment 2). After the candidate genes from three library screens were combined, a total of 114 genes in Experiment 1 and 128 genes in Experiment 2 met the corresponding criteria (see Supplementary Table S3). Seventeen genes were common to both experiments (Fig. 3A, also see Supplementary Table S4), and we tested cell viability using siRNA pools against these genes (Fig. 3B). We also tested the sensitivity to aurilide B upon siRNA-mediated knockdown of *prohibitin 1* (*PHB*). Among we have tested, siRNA-mediated knockdown of *ATP1A1* significantly sensitized cells to aurilide B. *ATP1A1* encodes the ubiquitously-expressed α-subunit of the Na^+^/K^+^ ATPase complex. Na^+^/K^+^ ATPase acts as the Na^+^/K^+^ pump at the plasma membrane to generate a Na^+^/K^+^ gradient, and thus plays an essential role in maintaining ionic homeostasis^17, 18^. Na^+^/K^+^ ATPase is also a signaling molecule, regulating the tyrosine kinase Src and phosphatidylinositide 3-kinase 1A, which in turn trigger downstream signaling events^19^. Na^+^/K^+^ ATPase is composed of catalytic α-subunits, auxiliary β-subunits, and regulatory γ-subunits^17, 18^. Our results suggest that ATP1A1 plays a critical role in determining sensitivity to aurilide B.

### Effect of ATP1A1 inhibition on sensitivity to aurilide B

**Figure 4:**
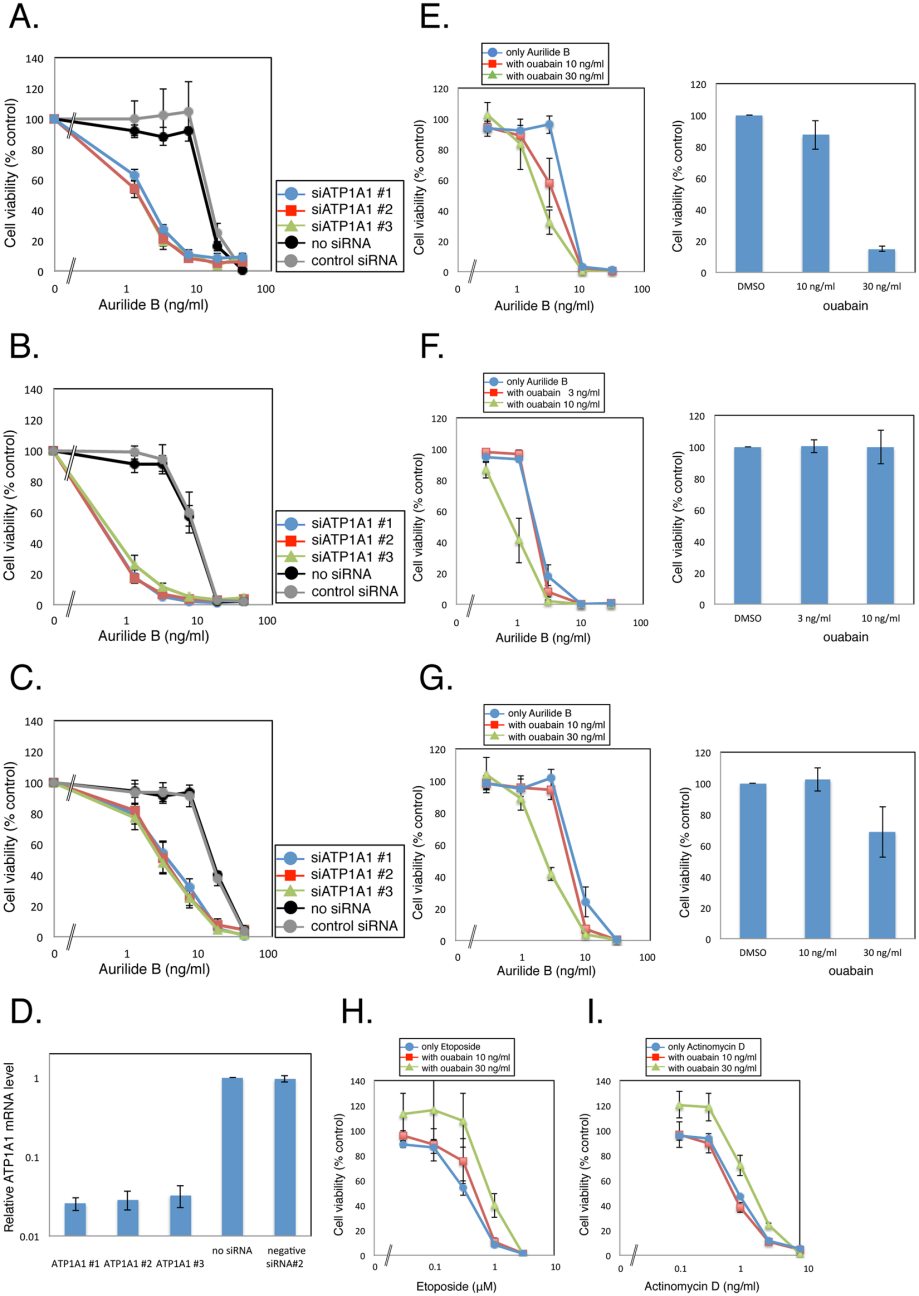
Effect of ATP1A1 inhibition on the sensitivity to aurilide B. (**A**–**C**) HeLa S3 (**A**), A549 (**B**) and HT-29 (**C**) cells were transfected with three independent siRNAs against ATP1A1, and then cultured for 2 days in the presence of various concentrations of aurilide B. Cell viability was measured by WST-8 assay. (**D**) HeLa S3 cells transfected with siRNAs against ATP1A1 were harvested, and the *ATP1A1* mRNA level was measured by qPCR. (**E**–**G**) HeLa S3 (**E**), A549 (**F**) and HT-29 (**G**) cells were treated for 4 days with the indicated concentrations of aurilide B in the presence of ouabain. Cell viability was measured by WST-8 assay. (**H**,**I**) HeLa S3 cells were treated for 4 days with the indicated concentrations of etoposide (**H**) and actinomycin D (**I**) in the presence of ouabain. Cell viability was measured by WST-8 assay. Data represent means ± SD from three independent experiments.

To confirm that ATP1A1 knockdown made cells more sensitive to aurilide B, we measured the aurilide B sensitivity of HeLa S3 cells treated with three individual siRNAs against ATP1A1, using WST-8 assays (Fig. 4A). Sensitivity to aurilide B was potentiated by all three siRNAs. We also found that the aurilide B sensitivity of lung cancer A549 cells and colon cancer HT-29 cells was potentiated by ATP1A1 siRNAs (Fig. 4B and C). Knockdown of ATP1A1 in cells transfected with siRNAs was verified by qPCR (Fig. 4D). Furthermore, to determine whether the potentiation of sensitivity to aurilide B is specific to ATP1A1, we investigated sensitivity to aurilide B upon treatment with siRNA pools against other subunit of Na^+^/K^+^ ATPase, as well as subunits of Ca^2+^ ATPase in HeLa S3 cells (see Supplementary Fig. S3A), as ATP2A2, one of the subunits of the latter, was also identified in the initial shRNA screening (Fig. 3B). The data showed that an siRNA pool against ATP1A3 sensitized cells to aurilide B. However, in HeLa S3 cells, only ATP1A1 is expressed among the α-subunit isoforms^20^. Therefore, we examined *ATP1A1* mRNA level in cells transfected with siRNAs against ATP1A1 and ATP1A3 (see Supplementary Fig. S3B and C). The result indicated that the expression level of ATP1A1 was decreased by transfection of siRNA against ATP1A3, suggesting a possible off-target effect on ATP1A1. We then used two individual siRNAs against ATP1A3 (see Supplementary Fig. S3D and E). Transfection of these ATP1A3 siRNAs did not potentiate the sensitivity to aurilide B or affect the *ATP1A1* mRNA levels. Thus, our results indicated that knockdown of ATP1A1 specifically sensitized cells to aurilide B. Next, to determine whether a Na^+^/K^+^ ATPase inhibitor could mimic the effect of ATP1A1 knockdown on aurilide B sensitivity, we measured cell viability upon treatment with both aurilide B and ouabain, a well-known Na^+^/K^+^ ATPase inhibitor that acts by targeting α-subunits^12, 13^. Combined treatment with ouabain sensitized HeLa S3 cells to aurilide B, although to a lesser extent than ATP1A1 knockdown (Fig. 4E). IC_50_ values were 8.37 ng/ml for aurilide B alone and 2.06 ng/ml for aurilide B with 30 ng/ml ouabain. Potentiation of the sensitivity to aurilide B was also observed with A549 and HT-29 cells (Fig. 4F and G). Moreover, we measured cell viability upon treatment with ouabain and other apoptosis inducers. Combined treatment with ouabain did not sensitize HeLa S3 cells to etoposide or actinomycin D (Fig. 4H and I). These results clearly demonstrate that knockdown or inhibition of ATP1A1 sensitized cells to aurilide B.

**Figure 5:**
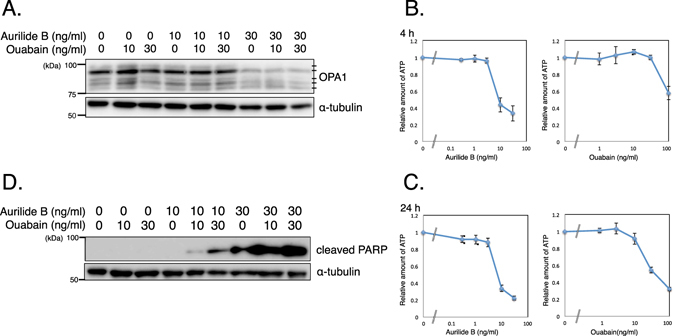
Combined treatment of aurilide B with ouabain. (**A**) Cells were treated for 24 h with the indicated concentrations of aurilide B or ouabain. OPA1 was analyzed by Western blotting. α-tubulin served as a loading control. (**B**,**C**) Cells were treated for 4 h (**B**) or 24 h (**C**) with the indicated concentrations of aurilide B or ouabain. Cellular ATP content was measured by ATPlite. For the 24-h samples, data were normalized against protein content. Data represent means ± SD from three independent experiments. (**D**) Cells were treated for 24 h with the indicated concentrations of aurilide B or ouabain. Cleaved PARP was analyzed by Western blotting. Uncropped blots are found in Supplementary Fig. S4.

Our results, together with those of previous reports, raise three possibilities. First, the action of aurilide B might affect the function of Na^+^/K^+^ ATPase. Aurilide B inhibits the interaction between prohibitin 1 and spastic paraplegia 7 (SPG7), a component of the protease complex involved in the processing of OPA1, resulting in the activation of SPG7^11^. Subsequently, activated SPG7 cleaves OPA1, which leads to mitochondrial fragmentation, poly (ADP-ribose) polymerase (PARP) cleavage, and cytochrome *c* release. Accordingly, we assessed the processing of OPA1 upon the treatment with aurilide B and ouabain (Fig. 5A). OPA1 was detected as five bands as reported previously^21, 22^ and the treatment with aurilide B showed selective loss of the long OPA1 isoforms. The treatment with ouabain did not affect the OPA1 processing. Next we examined the content of ATP in aurilide B-treated cells (Fig. 5B and C). When cells were treated with 10 ng/ml aurilide B, cellular ATP content decreased to 43% of control levels at 4 h and to 34% at 24 h, likely reflecting mitochondrial dysfunction. The Na^+^/K^+^ ATPase pump uses a considerable portion of the ATP produced in cells. Therefore, it is likely that in aurilide B–treated cells, the Na^+^/K^+^ ATPase pump cannot function properly due to the low ATP content, and it would be further disabled by knockdown or inhibition of the catalytic subunit ATP1A1, ultimately leading to cell death.

Second, inhibition of the Na^+^/K^+^ ATPase pump also induces mitochondria-mediated apoptosis. The cleavage of PARP by aurilide B was enhanced by ouabain (Fig. 5D). Treatment with 100 ng/ml ouabain also decreased the ATP content to 57% of control levels at 4 h and to 32% at 24 h (Fig. 5B and C). This is consistent with a previous report in which the authors also observed that fluorescently-labeled ouabain localizes to mitochondria in HeLa cells^23^. Ouabain induces Ca^2+^ oscillations^24^ and elevates the cytosolic Ca^2+^ level, which is important for ouabain-induced apoptosis^25, 26^. Recent work showed that knockdown of ATP1A1 induces mitochondrial dysfunction by disrupting ion homoeostasis, resulting in apoptosis^27^. Therefore, both treatments with aurilide B and knockdown or inhibition of ATP1A1 lead to mitochondria-mediated apoptosis, although it is not clear whether these treatments act on the same pathway or synergistically in parallel.

Third, our results do not rule out the possibility that potentiated sensitivity to aurilide B was mediated by knockdown or inhibition of ATP1A1 itself, rather than inhibition of Na^+^/K^+^ ATPase complex. To our knowledge, it is not likely that ATP1A1 plays a functional role apart from Na^+^/K^+^ ATPase complex, but given that more ATP1A1 molecules than β-subunits (ATP1B1 and ATP1B3) are present at the plasma membrane in HeLa cells^20^, it is not outside the realm of possibility that ATP1A1 has specific functions that are inhibited by ouabain. Notably, these three possibilities are not mutually exclusive. Further research that addresses these possibilities is underway.

In this study, we established a method for performing multiplex barcode sequencing following shRNA library screening. To obtain a proof of principle, we applied this approach to shRNA screening for candidate genes that modify the cytotoxicity of compounds with known targets. We identified ATP1A1 as a gene involved in survival of cells treated with aurilide B. Aurilide B has the cytotoxic effects in human leukemia, renal, and prostate cancer cell lines^10^. As reported previously using the NCI60 cancer cell panel, cardiac glycosides such as ouabain or digitoxin also has selective effects on different type of cancers^28^. Another work showed that digitoxin is significantly more toxic to melanoma cells than normal cells, which correlates well with the expression levels of ATP1A1 mRNA^27^. Therefore, combinatorial treatment with aurilide B and ouabain might trigger apoptosis in specific types of cancer cells with weaker effects to normal cells.

In addition to the identification of genetic components relevant to MoA of bioactive compounds, shRNA library have been used to profile genes that are essential for growth^16, 29, 30^ or synthetically lethal in cancer cells^31, 32^ and for various *in vivo* screening^33–35^. Moreover, recent development of genome-wide CRISPR/Cas9 libraries further extends the utility of chemical genetics for identification of genes relevant to compound targets and MoA^6, 36, 37^. A multiplexed sequence technique similar to the one used in this study was recently developed for identifying shRNA-encoding inserts of the TRC shRNA libraries that lack barcodes^38^. The use of indexed PCR primers when amplifying barcodes, or shRNA or guide RNA inserts, will facilitate unbiased functional genomics.

## Methods

### Cell culture

HeLa S3 cells were maintained in Dulbecco’s modified Eagle’s medium (DMEM) supplemented with 10% heat-inactivated fetal bovine serum (FBS), 100 units/ml penicillin, and 100 μg/ml streptomycin (Nacalai Tesque) at 37 °C under 5% CO_2_. A549 and HT-29 cells were maintained in RPMI1640 supplemented with 5% FBS, 0.1 mg/ml kanamycin, and antibiotic antimycotic solution (Sigma)^39^. Cell viability was measured by WST-8 (2-(2-methoxy-4-nitrophenyl)-3-(4-nitrophenyl)-5-(2, 4-disulfophenyl)-2H-tetrazolium) assay using Cell Count Reagent SF (Nacalai Tesque) on an iMark microplate reader (Bio-Rad Laboratories Inc.). Cellular ATP content was measured by ATPlite (PerkinElmer).

### Chemicals

Aurilide B was purified from cyanobacteria (*Okeania* sp.) collected in Chuuk, Federated States of Micronesia as follows: The frozen specimen (1040 g, wet weight) was extracted with MeOH. The extract was partitioned between H_2_O and CHCl_3_, and the H_2_O layer was further extracted with *n*-BuOH. The CHCl_3_ and *n*-BuOH layers were combined and fractionated by the Kupchan procedure^40^ to yield *n*-hexane, CHCl_3_, and 60% MeOH layers. The obtained CHCl_3_ layer was concentrated and separated by octadecylsilane (ODS) flash column chromatography and followed by ODS HPLC to give 1.6 mg of aurilide B. The structure of aurilide B was confirmed by comparison of spectral data such as ESI-MS and ^1^H NMR with those of previous literature^10^. Etoposide (Sigma), actinomycin D (Sigma) and ouabain (g-strophanthin; Merck) were purchased from the indicated suppliers.

### Lentiviral shRNA screening

DECIPHER^TM^ barcoded shRNA libraries, Human Modules 1, 2 and 3, were obtained from Cellecta. Packaging into lentiviral particles using 293LTV cells was performed as described previously^41^. HeLa S3 cells (1 × 10^7^ cells at a minimum) were transduced with each of the lentiviral shRNA libraries at 50% efficiency in DMEM containing 10% FBS, 2 mM L-alanyl-L-glutamine (Nacalai Tesque) and 5 μg/ml Polybrene (Sigma). After 24 h, the medium containing viral particles was replaced with fresh medium. After an additional 24 h, the infected cells were selected with 2 μg/ml puromycin for 48 h. The cells were then split, and aliquots were treated or untreated with the indicated compounds. The cells were passaged as necessary to maintain exponential growth in the presence or absence of the compounds for 6 to 10 days, and then harvested. For comparison shown in Fig. 1A, an aliquot of untreated cell pellets was sent to Cellecta for DNA extraction, PCR amplification of barcodes, and barcode quantitation by next-generation sequencing.

### Multiplex barcode sequencing

Genomic DNA was prepared from the cells as described in the user manual for the Cellecta DECIPHER shRNA library. The fragments for barcode sequencing were amplified from genomic DNA with two rounds of PCR: the first round was 10 cycles with FwdHTS and RevHTS primers, which were designed by Cellecta; and the seond round was 16 cycles with FwdGex and RevGex-index-GexSeqN (5′-AATGATACGGCGACCACCGAGATCTACAC- index-ACACTCTTTCCCTACACGACGCTCTTCCGATCTNNNNACAGTCCGAAACCCCAAACGCACGAA-3′). The 8-bp index sequences, designed by Illumina (Oligonucleotide sequences © 2007–2012 Illumina, Inc. All rights reserved.), were as follows: index 1, 5′-TAGATCGC-3′; index 2, 5′-CTCTCTAT-3′; index 3, 5′-TATCCTCT-3′; and index 4, 5′-AGAGTAGA-3′. The resultant PCR products were separated on agarose gels, purified using the QIAquick Gel Extraction Kit (Qiagen), and quantified using the Library Quantification Kit (KAPA). Equal amounts of four PCR products with different indexes were mixed in a single lane of an Illumina HiSeq2500 flow cell. Sequencing and barcode deconvolution and enumeration were performed at the GeNAS facility of our institute (Yokohama, Japan). During the de-multiplex process of indexes to obtain the results shown in Fig. 1A and B, 92.4% and 91.8% of the clusters were categorized by each index, and 7.6% and 8.2% of the clusters were not categorized, respectively. To evaluate quantitative amplification, 0.06, 0.18, 0.54, and 1.62 fg of each of three DNA spike-ins harboring designed barcodes and surrounding vector sequences (see Supplementary Table S2; Integrated DNA Technologies, Coralville, IA) were added to 200 μg of genomic DNA obtained from cells transduced with the Human Module 1 library, prior to PCR amplification (Fig. 1C). For each sample, total counts of reads were normalized to 20 million. The normalized read count of each shRNA from aurilide B-treated samples was divided by that from untreated samples to obtain a fold change (aurilide B-treated/untreated). We set the cutoff values so as to select less than 1% of the total number of screened genes (~15,000) as candidates. Genes passing this filter criteria with at least two shRNAs were selected as candidates. Human modules 1, 2 and 3 share multiple shRNAs targeting 10 control genes, which have more statistical chances to be selected. These control genes were excluded from the candidates.

### siRNA screening

HeLa S3 cells were transfected with 10 nM siRNA pool (Human siGENOME SMARTpool, GE Dharmacon), which contains four oligos against each gene, using Lipofectamine RNAiMAX reagent (Thermo Fisher Scientific). As negative controls, siGENOME Non-Targeting siRNA #2 (GE Dharmacon) and Select Negative Control No. 2 siRNA (Ambion) were used. Three different siRNAs against *ATP1A1* (#1, s1718; #2, s1719; and #3, s1720) and two different siRNAs against *ATP1A3* (#1, s1724; and #2, s1725) were purchased from Ambion. After 48 h, transfected cells were split and seeded in 96-well plates, and then treated with the indicated concentrations of etoposide or aurilide B for 48 h.

### Quantitative PCR

Total RNA from cells was prepared using the RNeasy Mini Kit (Qiagen), and cDNA was synthesized using the PrimeScript RT reagent Kit (Takara Bio Inc.). Expression of target genes was analyzed using SYBR Premix Ex Taq II (Takara Bio Inc.) on a Thermal Cycler Dice Real Time System (Takara Bio Inc.). Primers for *ATP1A1* were 5′-ACAGACTTGAGCCGGGGATTA-3′ (forward) and 5′-TCCATTCAGGAGTAGTGGGAG-3′ (reverse). Primers for *GAPDH*, used as a reference, were 5′-GCACCGTCAAGGCTGAGAAC-3′ (forward) and 5′-TGGTGAAGACGCCAGTGGA-3′ (reverse).

### Western blotting

Cells were lysed in lysis buffer containing 50 mM Tris-HCl (pH 7.5), 150 mM NaCl, 5 mM EDTA, 0.1% NP-40, 1 mM dithiothreitol, and protease inhibitor cocktail (Roche). Proteins were separated by sodium dodecyl sulfate–polyacrylamide gel electrophoresis (SDS-PAGE) and transferred to a polyvinylidene difluoride membrane (Millipore) by electroblotting. After the membranes were incubated with primary and secondary antibodies, the immune complexes were detected with the Immobilon Western Chemiluminescent HRP Substrate (Millipore), and luminescence was analyzed on a Luminescent Image Analyzer LAS-4000 mini (Fujifilm). Primary antibodies used are anti-OPA1 (Cell signaling technology), anti-cleaved PARP (Cell signaling technology) and anti-α-tubulin (Sigma). Horseradish peroxidase–conjugated anti-mouse IgG and anti-rabbit IgG secondary antibodies were purchased from GE Healthcare.

## Electronic supplementary material

Supplementary Information
