# Supplementary material for: A quantitative shRNA screen identifies ATP1A1 as a gene that regulates cytotoxicity by aurilide B
Source: Sci Rep. 2017 May 17;7:2002. doi: 10.1038/s41598-017-02016-4 (PMC5435677; doi:10.1038/s41598-017-02016-4)
Supplement: Supplementary file 1 — Supplementary Information [file 41598_2017_2016_MOESM1_ESM.pdf]

Supplementary information

A quantitative shRNA screen identifies *ATP1A1* as a gene that regulates cytotoxicity by aurilide B

Shohei Takase, Rumi Kurokawa, Daisuke Arai, Kind Kanemoto Kanto, Tatsufumi Okino, Yoichi Nakao, Tetsuo Kushiro, Minoru Yoshida, and Ken Matsumoto

Figure S1. Scheme of barcode amplification

Barcode amplification protocols: left panel, as recommended by Cellecta; right panel, with indexed tagged primer, as described in this study.

Figure S2. shRNA screening with aurilide B in HeLa S3 cells under two independent conditions.

HeLa S3 cells were infected with Module 1 and split into two subpopulations, one of which was treated with 6 ng/ml aurilide B for 10 days (Exp. 1) or 10 ng/ml aurilide B for 7 days (Exp. 2). Relative read counts of each barcode were compared between treated and untreated cells. shRNAs against ATP1A1 are highlighted in red.

Figure S3. Effect of knockdown of genes encoding Na^+/K^+ ATPase or Ca^{2+} ATPase complex on sensitivity to aurilide B

(A) HeLa S3 cells were transfected with siRNA pools against genes encoding components of the Na^+/K^+ ATPase or Ca^{2+} ATPase complex, and then cultured for 2 days in the presence of the indicated concentrations of aurilide B. Cell viability was measured by WST-8 assay. (B and C) Cells transfected with siRNA pools against ATP1A1 (B) and ATP1A3 (C) were harvested, and the *ATP1A1* mRNA level was measured by qPCR. (D) HeLa S3 cells were transfected with individual siRNAs against ATP1A3 and then cultured for 2 days in the presence of the indicated concentrations of aurilide B. Cell viability was measured by WST-8 assay. (E) Cells transfected with siRNAs against ATP1A3 were harvested, and the *ATP1A1* mRNA level was measured by qPCR. Data

represent means ± SD from three independent experiments.

Figure S4. Uncropped images for Figure 5A and D.

The chemiluminescent signals were recorded using a LAS 4000 mini instrument and the ImageQuant LAS 4000 software. For Figure 5D, after detecting the OPA1 levels, the antibodies bound on the membrane were removed and then incubated with blocking solution containing anti-α-tubulin antibody.

Table S1. Number of shRNAs and genes categorized by the ratio of barcode read counts between non-indexed and indexed PCR methods

shRNAs were categorized by fold change (indexed sequencing/non-indexed sequencing), and genes with two or more shRNAs in each category were counted. For the right two columns, barcodes with ≤ 100 reads were filtered out for quality assurance, and then shRNAs and genes were categorized as above.

Table S2. Sequences of DNA spike-ins

Table S3. Genes selected by shRNA screening as candidates relevant to aurilide B sensitivity

Genes with two or more shRNAs with fold change (FC, Aurilide B-treated/Untreated) < 0.4 (Exp. 1) or 0.33 (Exp. 2) are listed.

Table S4. 17 candidate genes identified in two independent shRNA screens

Takase et al., Fig. S1

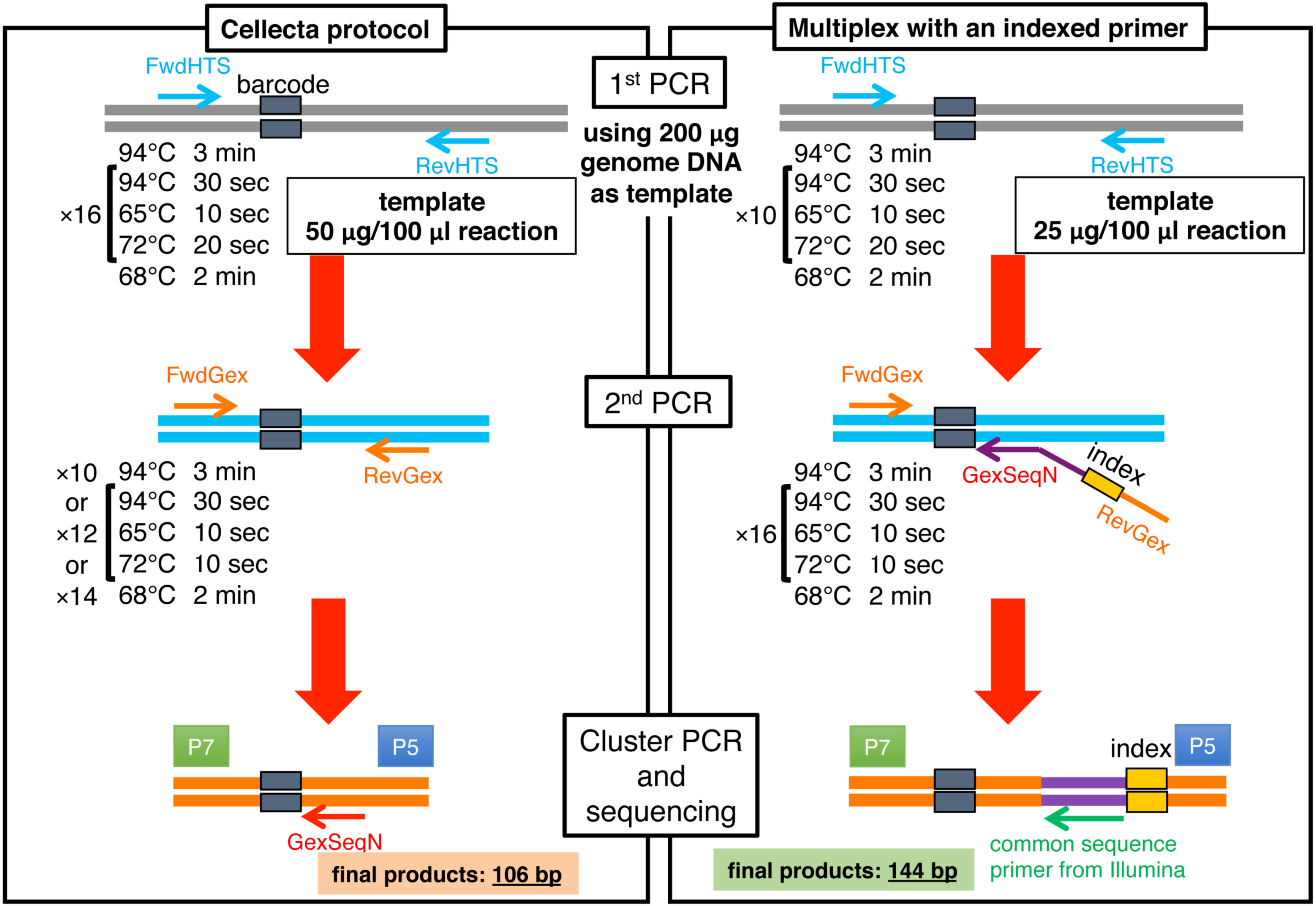

Takase et al., Fig. S2

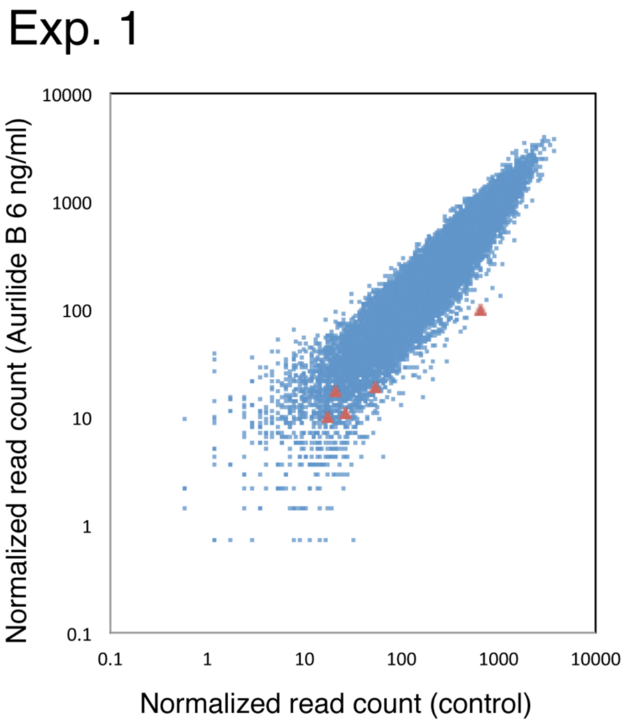

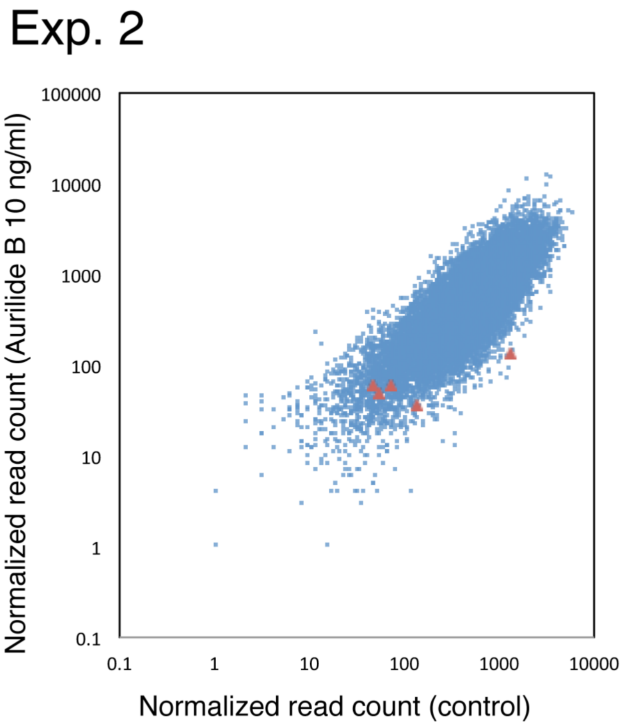

Takase et al., Fig. S3

A.

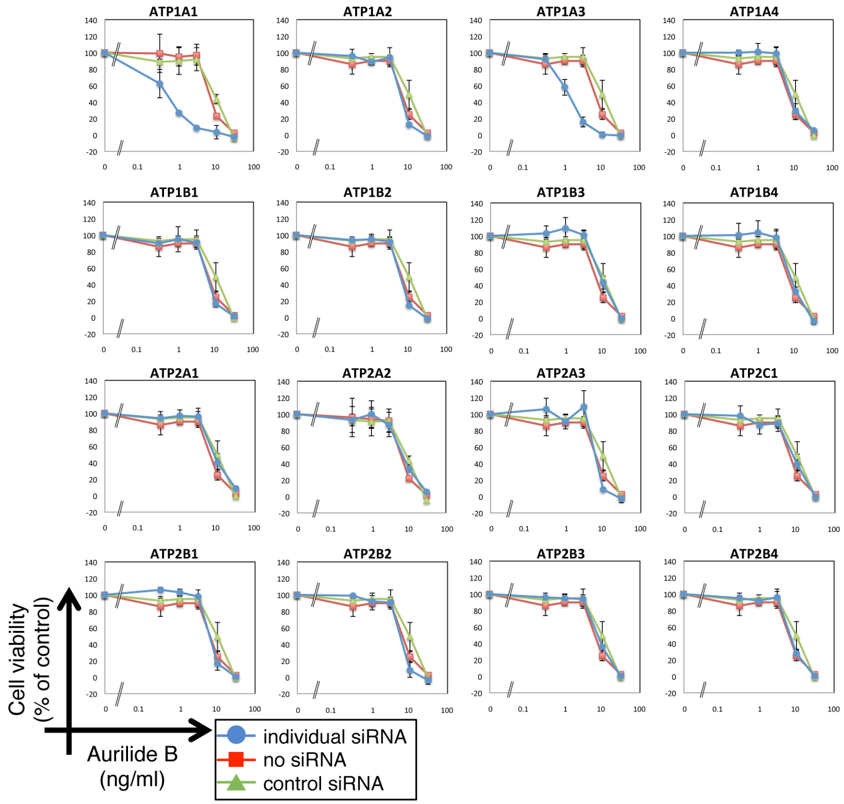

B.

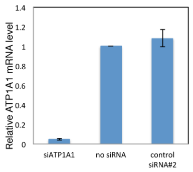

C.

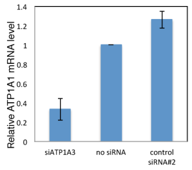

D.

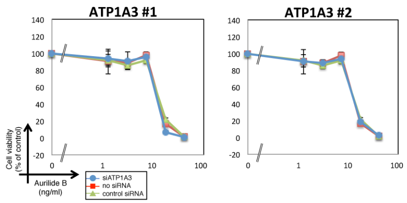

E.

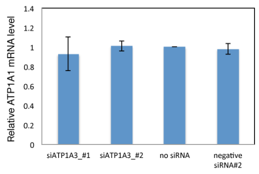

Takase et al., Fig. S4

Figure 5A

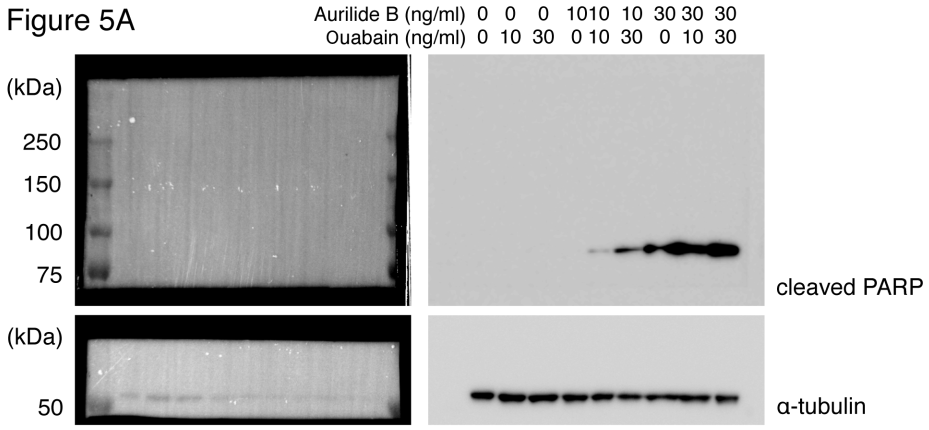

Figure 5D

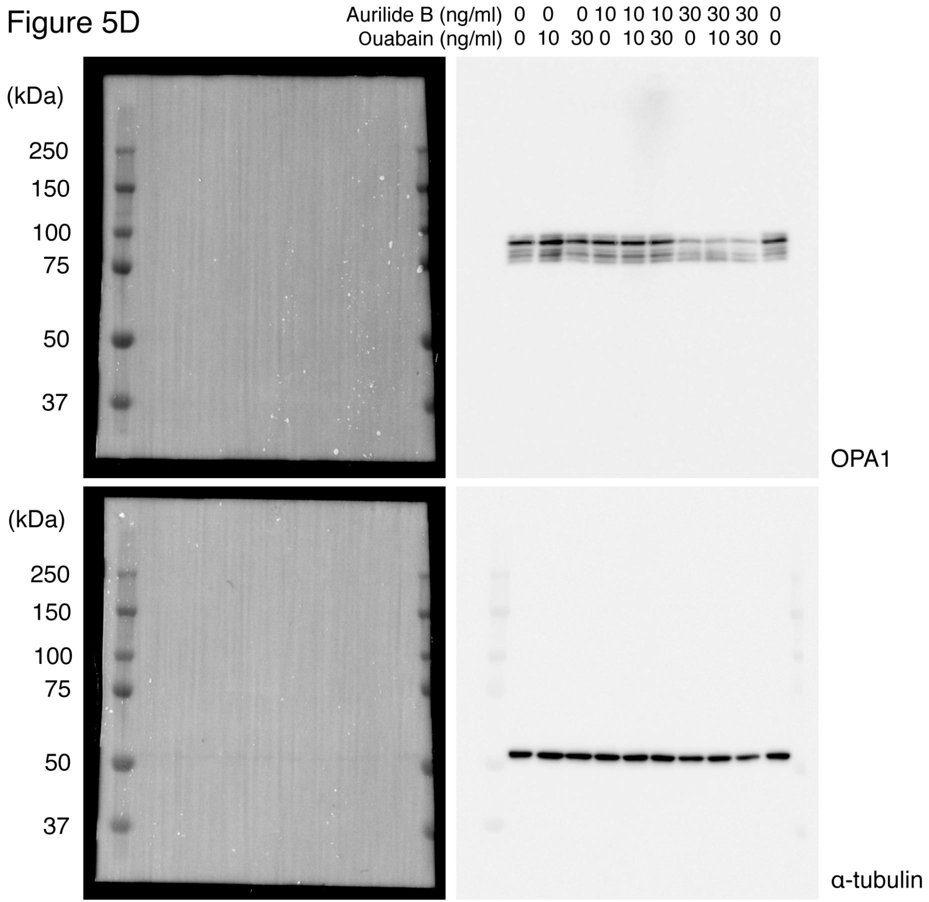

Takase et al., Table. S1

Fold change (indexed/non-indexed)	no cut-off		cut-off ≦100 read count (indexed-seq)	
	shRNA count	Gene count (freq ≧2 shRNAs)	shRNA count	Gene count (freq ≧2 shRNAs)
≧2	409	27	54	0
1~2	14299		13089	
0.5~1	12474		11795	
≦0.5	262	19	81	0
total shRNAs	27444	5046	25019	
cut-off shRNAs	0		2425	

Sequences of DNA spike-ins (Related to Experimental Procedures)

Name	Sequence (Designed barcodes are underlined)
Spike_HM1_1	GAAGTTAATATTCATAGCTTCACGCTCGATCTCAAAGGCTTTTTTGGCAAGCAAAAGACGGCATACGA<br>GATATATCGGTACCTAGGTACATTTCGTGCGTTTGGGGTTTCGGACTGTAGAACTCTGAACCTCTC<br>GGTGGTCGCCGTATCATTAGAATTCTCGACCTCGAGACAAATGGCAGTATTCATCCACAATTTTAA
Spike_HM1_2	GAAGTTAATATTCATAGCTTCACGCTCGATCTCAAAGGCTTTTTTGGCAAGCAAAAGACGGCATACGA<br>GATATGTCATAGCTGCTAGTGCTTTCGTGCGTTTGGGGTTTCGGACTGTAGAACTCTGAACCTCTC<br>GGTGGTCGCCGTATCATTAGAATTCTCGACCTCGAGACAAATGGCAGTATTCATCCACAATTTTAA
Spike_HM1_3	GAAGTTAATATTCATAGCTTCACGCTCGATCTCAAAGGCTTTTTTGGCAAGCAAAAGACGGCATACGA<br>GATATCGATCGACTGGTCGATCGTTCGTGCGTTTGGGGTTTCGGACTGTAGAACTCTGAACCTCTC<br>GGTGGTCGCCGTATCATTAGAATTCTCGACCTCGAGACAAATGGCAGTATTCATCCACAATTTTAA

Takase et al., Table. S3

Exp. 1
6 ng/ml
10 days
FC≦0.4
≧2 shRNAs
114 genes

AP2S1	HARS2	RPL34	TRIM61
APBA1	HDAC3	RPL35A	TTC15
ARL17A	HNF4A	RPL38	TTC27
ARSB	HNRNPU	RPL6	TTC38
ATP1A1	INTS8	RPL8	TUBGCP3
ATP1B1	ITGB4BP	RPS23	UBE2D3
ATP2A2	JAKMIP1	RPS24	UBE2M
BANP	LOC374443	RPS4X	UTP14C
BEND6	LYPD1	SCML1	VASH2
C13ORF15	MAGEA10	SDAD1	WNT7A
C19orf29	MED20	SEC22B	YKT6
CALR	MLF1IP	SERPINA10	ZBTB34
CCNK	MRPS18B	SF3A1	ZBTB47
CCT4	MYH8	SF3B2	ZMYND12
CCT6A	N4BP2L2	SF3B3	ZNF260
CD68	NARS	SF3B5	ZNF274
CLEC1A	NKX2-3	SFRS8	ZNF451
CTSH	NMUR2	SH3BP5	ZNF558
CXCL11	PCDH1	SHFM1	
CXorf15	PGCP	SKAP2	
DYNC1H1	PI16	SMAD2	
ECSIT	PRKX	SNRPF	
EFTUD1	PRRX2	SON	
EIF2B5	PSMA6	SP1	
EIF6	PSMB7	SP6	
ERBB2IP	PSMD11	SRP72	
ERCC3	PSMD7	SUPV3L1	
EXOSC5	PWP2	TAGLN3	
FBL	PYDC1	TBC1D10A	
FHOD1	RETN	TGIF2LY	
FXYD5	RIMS3	TMEM187	
GRIA3	RPL11	TNFAIP8	

Exp. 2
10ng/ml
7 days
FC≦0.33
≧2 shRNAs
128 genes

ALG1	COL21A1	IDH3A	RGPD4
ANAPC10	COL6A6	IFNA21	RRM1
ANP32A	CRLF2	IL18BP	SFN
ANP32B	CSF1	KCNH7	SLC22A6
AP2M1	CSH1	KCNJ5	SLC25A22
AP2S1	CTNNB1	KDM3A	SLC34A1
APH1B	CYP7A1	KIF23	SNRPD2
APPBP2	DDX3X	LCP2	SNX4
ARHGEF6	DEF6	LOC284998	SPOCD1
ARL17A	DKKL1	LYST	STX6
ARSB	DPPA2	MAGI3	STXBP4
ATP1A1	DPT	MCM9	STYXL1
ATP2A2	E2F1	MPP1	TAS2R5
ATP2B3	ECSIT	MTA2	TBC1D29
ATP6V0C	ENTPD1	NCOR1	TDG
ATXN7L3	ERBB2IP	NCR2	THOC2
B3GALT6	ERCC3	NKD2	TMEM187
BCL2L1	ESRRA	NLK	TNFRSF10A
BP75	F10	NUP88	TNFSF8
C13ORF15	FBXL8	OSBP	TSC1
C1orf51	FBXW7	PIGO	TSG101
C6orf118	FHOD1	PIK3CA	TSPAN3
CCL19	FRAT2	PIK3R1	UBE2N
CCL20	FXYD5	PIP4K2C	UBE2NL
CD68	GJA10	PLD3	UNC50
CDC16	GM2A	PODN	VASH2
CDC27	GPD2	PPDPF	ZBTB12
CERCAM	GPR81	PPY2	ZBTB47
CKLF	GSS	PTAFR	ZC3H4
CLEC1A	GTF2H5	RAPSN	ZFP36
COL13A1	HELB	REG3G	ZNF260
COL16A1	HSBP1	RERE	ZNF670

Takase et al., Table. S4

Module	RefSeq	Gene Symbol	Description
3	NM_004069.3	AP2S1	adaptor related protein complex 2 sigma 1 subunit
3	NM_001113738.1	ARL17A	ADP ribosylation factor like GTPase 17A
1	NM_198709.1	ARSB	arylsulfatase B
1	NM_000701.6	ATP1A1	ATPase Na+/K+ transporting subunit alpha 1
1	NM_170665.2	ATP2A2	ATPase sarcoplasmic/endoplasmic reticulum Ca2+ transporting 2
1	NM_014059.2	C13ORF15	regulator of cell cycle
1	NM_001251.2	CD68	CD68 molecule
3	NM_016511.2	CLEC1A	C-type lectin domain family 1 member A
1	NM_016581.2	ECSIT	ECSIT signalling integrator
3	NM_001006600.1	ERBB2IP	erbb2 interacting protein
1	NM_000122.1	ERCC3	ERCC excision repair 3, TFIIH core complex helicase subunit
3	NM_013241.2	FHOD1	formin homology 2 domain containing 1
2	NM_001164605.1	FXYD5	FXYD domain containing ion transport regulator 5
3	NM_003492.2	TMEM187	transmembrane protein 187
3	NM_001136474.1	VASH2	vasohibin 2
3	NM_145166.3	ZBTB47	zinc finger and BTB domain containing 47
3	NM_001012756.2	ZNF260	zinc finger protein 260
